# Supplementary material for: Tightly-orchestrated rearrangements govern catalytic center assembly of the ribosome
Source: Nat Commun. 2019 Feb 27;10:958. doi: 10.1038/s41467-019-08880-0 (PMC6393466; doi:10.1038/s41467-019-08880-0)
Supplement: Supplementary file 1 — Supplementary Information [file 41467_2019_8880_MOESM1_ESM.pdf]

Tightly-orchestrated rearrangements govern catalytic center assembly of the ribosome

Zhou et al.

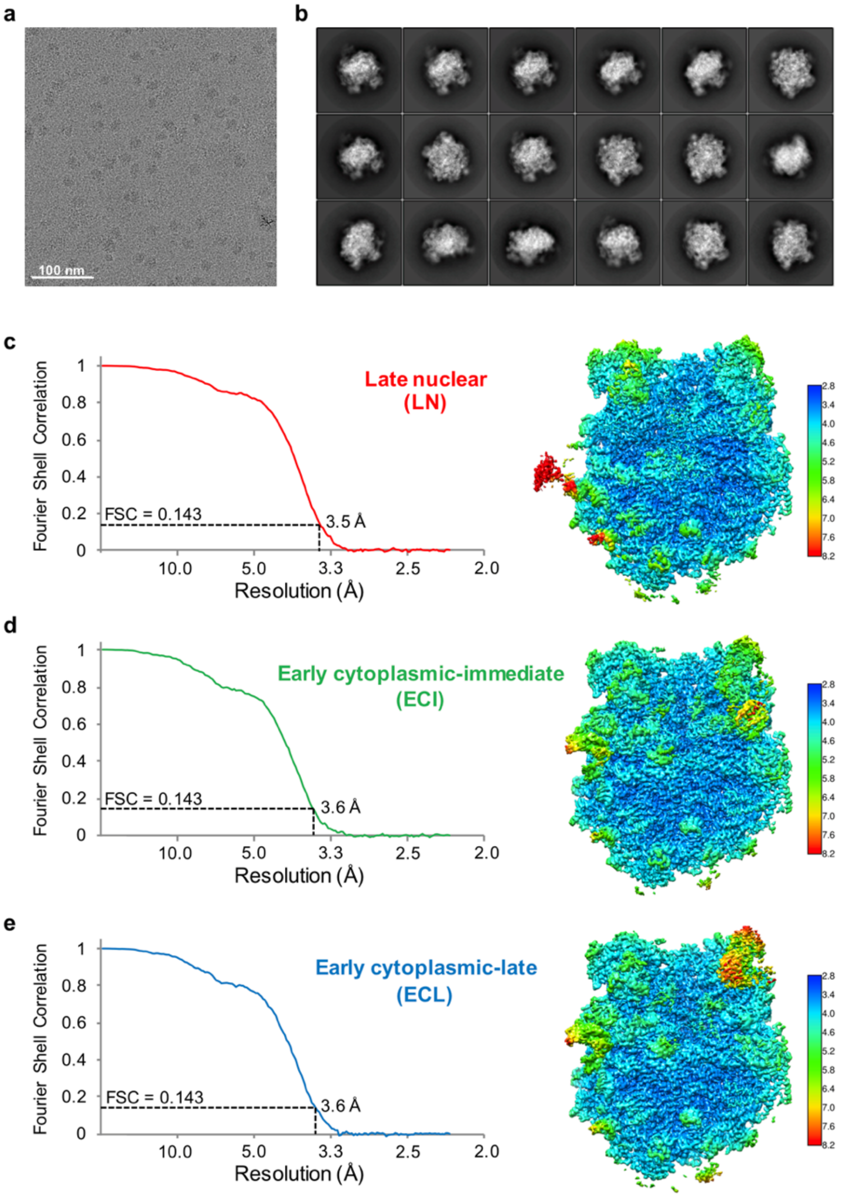

Supplementary Fig. 1 | Cryo-EM structure determination of the Rlp24ΔC-TAP pre-60S ribosomes. a, A drift-corrected cryo-EM micrograph of the Rlp24ΔC-TAP pre-60S ribosomes imaged on a Gatan K2 Summit direct electron detector. The scale bar indicates 100 nm. **b,** Reference-free 2D class averages of the Rlp24ΔC-TAP pre-60S particles. The width of the boxes is 422.4 Å. **c,** Fourier shell correlation curve (left) and local resolution map (right) for the late nuclear (LN) pre-60S particles. The overall resolution for the map is 3.5-Å based on the 0.143 FSC criterion. **d,** Fourier shell correlation curve (left) and local resolution map (right) for the early cytoplasmic-immediate (ECI) pre-60S particles. The overall resolution for the map is 3.6-Å based on the 0.143 FSC criterion. **e,** Fourier shell correlation curve (left) and local resolution map (right) for the early cytoplasmic-late (ECL) pre-60S particles. The overall resolution for the map is 3.6-Å based on the 0.143 FSC criterion. The release of the NTD of Nog1 from the A site likely causes high flexibility of the P stalk, leading to badly resolved Rpl12 (uL11) and Mrt4 on the P stalk.

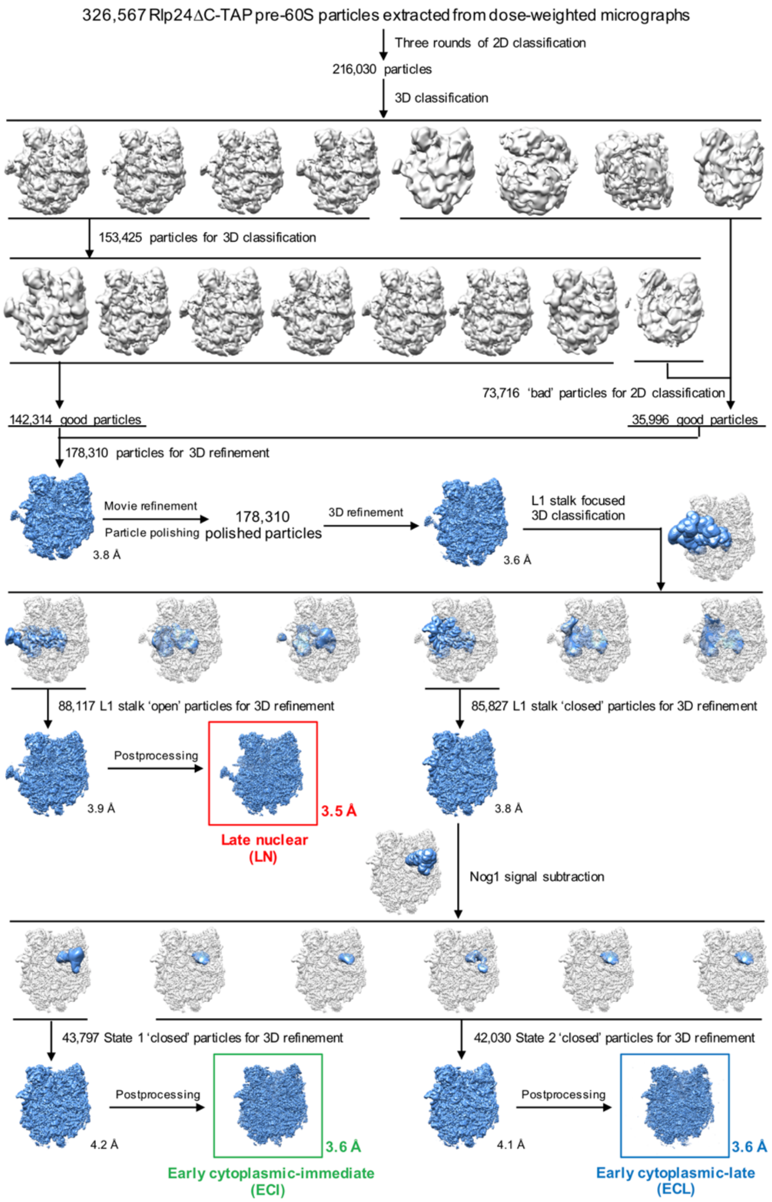

Supplementary Fig. 2 | Classification and refinement workflow of the Rlp24ΔC-TAP pre-60S ribosomes. A total starting stack of 326,567 Rlp24ΔC-TAP particles extracted from dose-weighted micrographs were subjected to three rounds of reference-free 2D classification. A subset of 216,030 particles from the final 2D classes were selected and subjected to two rounds of 3D classification and an additional round of 2D classification, resulting in 178,310 'good' particles which were further subjected to 3D refinement, movie refinement and particle polishing in RELION. Next, the polished particles were subjected to L1 stalk focused 3D classification using a soft mask around the L1 stalk, resulting in 88,117 L1 stalk 'open' particles and 85,827 L1 stalk 'closed' particles. The L1 stalk 'open' particles, which were termed late nuclear (LN) particle in this paper, were further analyzed in RELION, leading to a 3.9-Å map after 3D refinement and a 3.5-Å map after postprocessing. The L1 stalk 'closed' particles, which were termed early cytoplasmic (EC) particles in this paper, were further subjected to signal subtraction analysis using a soft mask around the Nog1 N-terminal four-helical-bundle domain and GTPase domain (NTD-GD), resulting in two subsets of particles: 43,797 particles with clear density of NTD-GD (early cytoplasmic-immediate (ECI)) and 42,030 particles with no density of NTD-GD (cytoplasmic-late (ECL)). Further analysis of the ECI particles led to a 4.2-Å 3D refinement map and a 3.6-Å postprocess map, where the NTD-GD of Nog1 is in its canonical position with the N-terminus of Nmd3 being unresolved. In contrast, further analysis of the ECL particles led to a 4.1-Å 3D refinement map and a 3.6-Å postprocess map, where Nog1 NTD-GD has been released from the A site while the N-terminus of Nmd3 has docked onto Tif6.

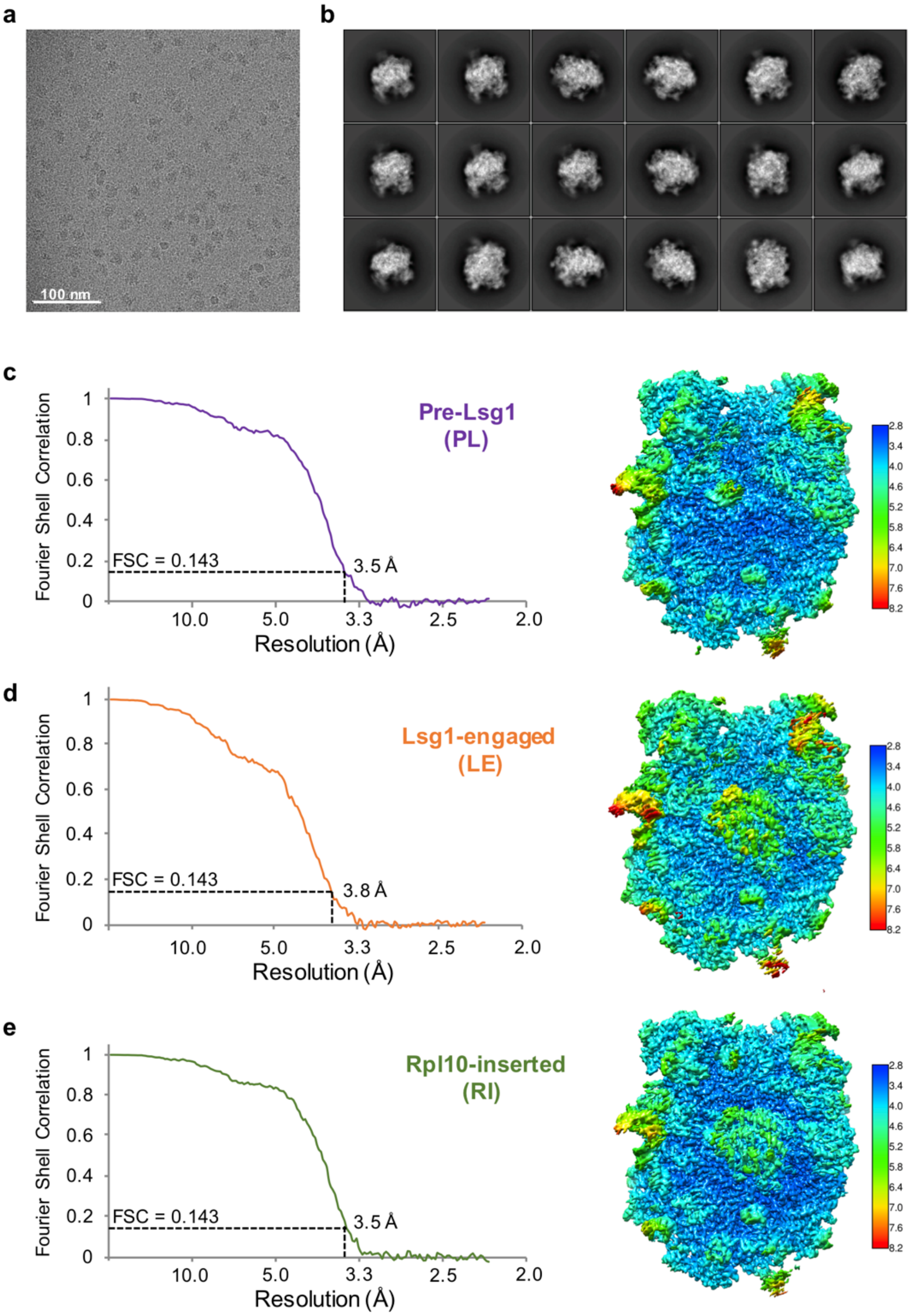

Supplementary Fig. 3 | Cryo-EM structure determination of the Nmd3-TAP pre-60S ribosomes from diazaborine-treated cells. a, A drift-corrected cryo-EM micrograph of the Nmd3-TAP pre-60S ribosomes imaged on a Gatan K2 Summit direct electron detector. The scale bar indicates 100 nm. **b,** Reference-free 2D class averages of the Nmd3-TAP pre-60S particles. The width of the boxes is 422.4 Å. **c,** Fourier shell correlation curve (left) and local resolution map (right) for the pre-Lsg1 (PL) pre-60S particles. The overall resolution for the map is 3.5-Å based on the 0.143 FSC criterion. **d,** Fourier shell correlation curve (left) and local resolution map (right) for the Lsg1-engaged (LE) pre-60S particles. The overall resolution for the map is 3.8-Å based on the 0.143 FSC criterion. **e,** Fourier shell correlation curve (left) and local resolution map (right) for the Rpl10 (uL16)-inserted (RI) pre-60S particles. The overall resolution for the map is 3.5-Å based on the 0.143 FSC criterion.

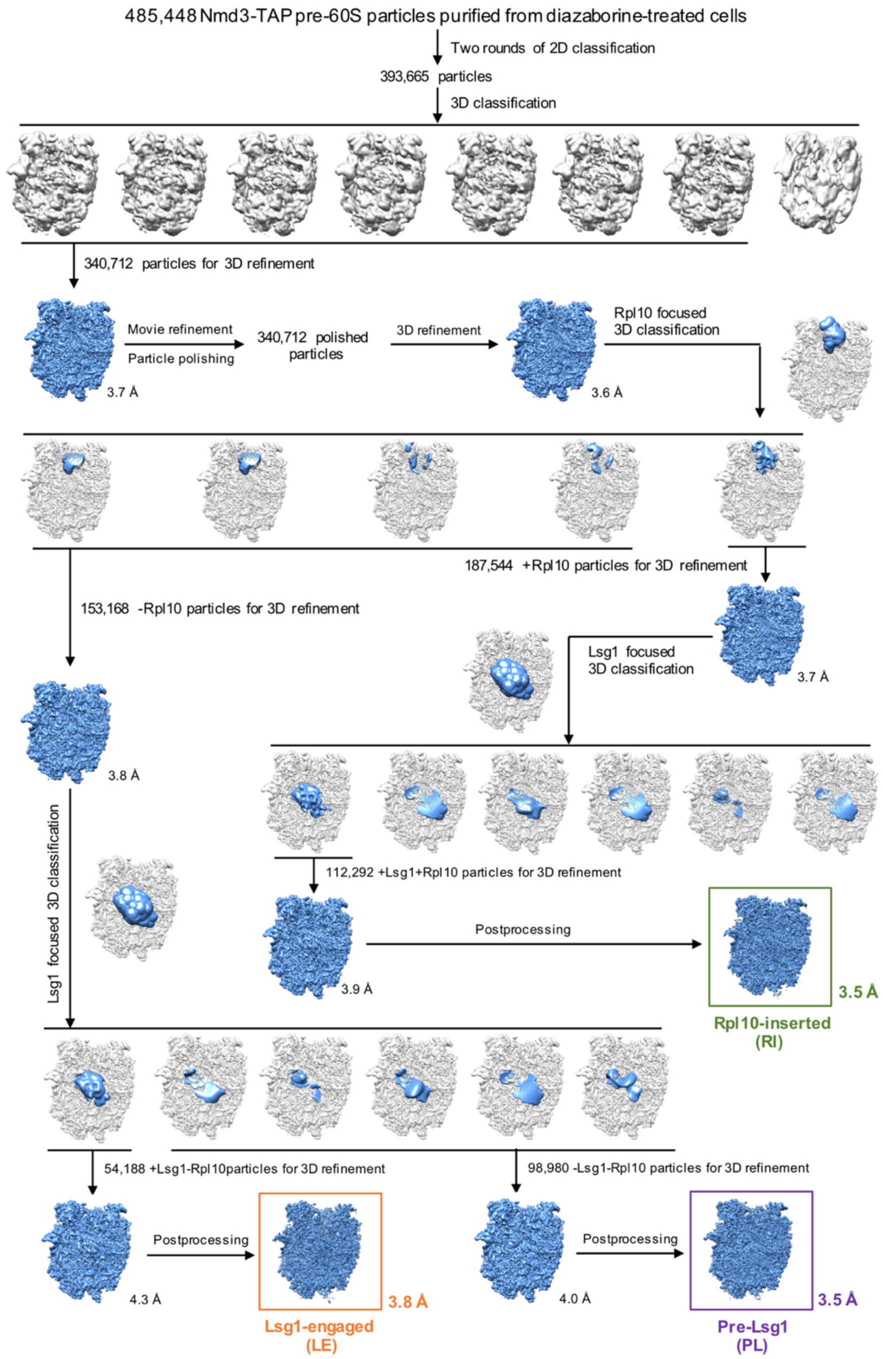

Supplementary Fig. 4 | Classification and refinement workflow of the Nmd3-TAP pre-60S ribosomes purified from diazaborine-treated cells. A total starting stack of 485,448 particles were subjected to two rounds of reference-free 2D classification. A subset of 393,665 particles from the final 2D classes were selected and subjected to 3D classification, resulting in 340,712 'good' particles which were further subjected to 3D refinement, movie refinement and particle polishing in RELION. The polished particles were first subjected to focused 3D classification with a soft mask around Rpl10 (uL16), resulting in 153,168 particles without Rpl10 (-Rpl10) and187,544 particles with Rpl10 (+Rpl10). The -Rpl10 particles were further subjected to Lsg1 focused 3D classification, resulting in 98,980 -Lsg1-Rpl10 particles and 54,188 +Lsg1-Rpl10 particles, which were termed pre-Lsg1 (PL) and Lsg1-engaged (LE) particles, respectively. Further processing of the PL particles led to a 4.0-Å 3D refinement map and a 3.5-Å postprocess map, while further processing of the LE particles resulted in a 4.3-Å 3D refinement map and a 3.8-Å postprocess map. The +Rpl10 particles were also subjected to Lsg1 focused 3D classification, resulting in 112,292 particles with strong Lsg1 density (+Lsg1+Rpl10), which were named Rpl10-inserted (RI) particles. Further processing of the RI particles led to a 3.9-Å 3D refinement map and a 3.5-Å postprocess map.

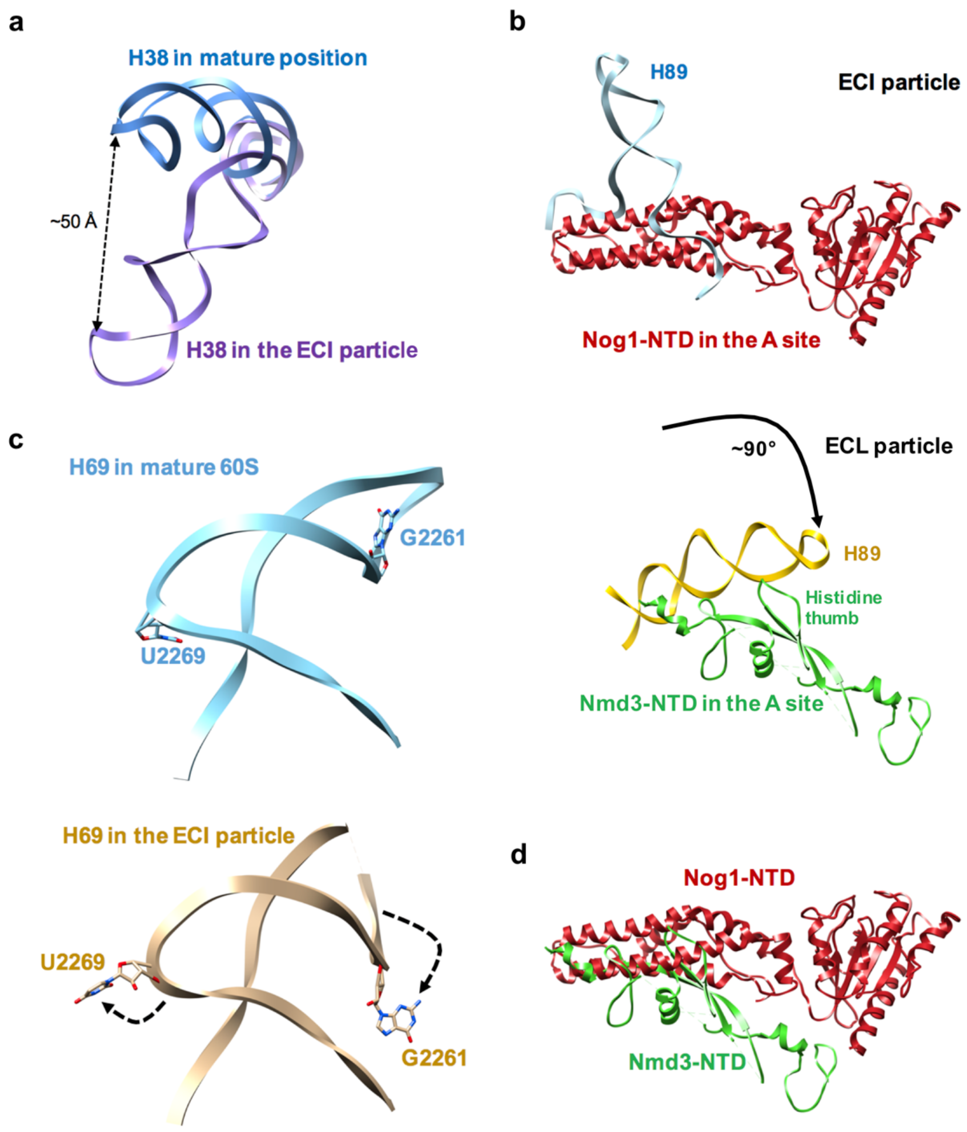

Supplementary Fig. 5 | Dynamics of H38, H89 and H69 in pre-60S ribosomes. a, Comparison of H38 in its mature position (PDB: 4V88) [1] and H38 from the early cytoplasmic-immediate (ECI) particle. There is a ~50 Å shift of the H38 tip. **b,** Rearrangement of H89 in transition from ECI (top) to ECL (bottom). Upon the release of Nog1 NTD from the A site and Nmd3 NTD docking onto Tif6, H89 is rearranged to its near-mature position by rotating about ~90° to interact with the histidine thumb of Nmd3. **c,** Comparison of H69 in mature 60S (top) (PDB: 4V88) [1] and H69 from the early cytoplasmic-immediate (ECI) particle (bottom). G2261 and U2269 are flipped out in the ECI particle relative to mature 60S. The extreme tip of H89 in the ECI particle is not modeled due to bad density. **d,** Structural conflict of Nog1 and Nmd3 on pre-60S particles. Nog1 NTD from the ECI particle and Nmd3 NTD from the early cytoplasmic-late (ECL) particle are overlaid.

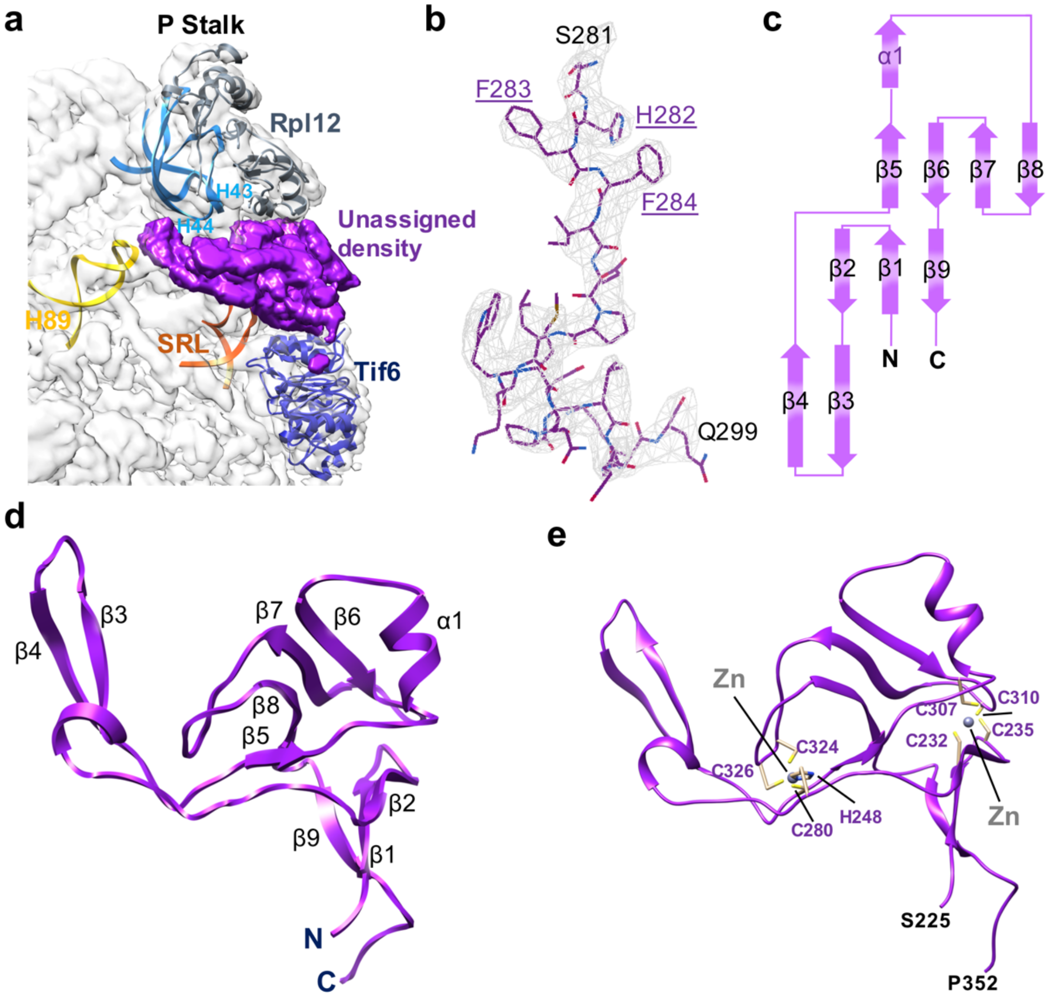

Supplementary Fig. 6 | The atomic structure of the zinc-binding domain of Yvh1. a, Segmented unassigned density (purple) positioned between Tif6 and the P stalk in the electron density map of the pre-Lsg1 particle with relevant elements of the 60S subunit indicated. SRL, sarcin-ricin loop. **b,** Example of the quality of the electron density map showing fitting of side chains of Yvh1 from residues S281 to Q299. Residues H282, F283 and F284 were used as our starting register for model building. **c,** Topology of the zinc-binding domain of Yvh1. **d,** Atomic structure of the zinc-binding domain of Yvh1. Secondary structure elements are indicated. **e,** Detail of the two zinc centers of Yvh1 with coordinating residues indicated and zinc ions modeled in their predicted locations.

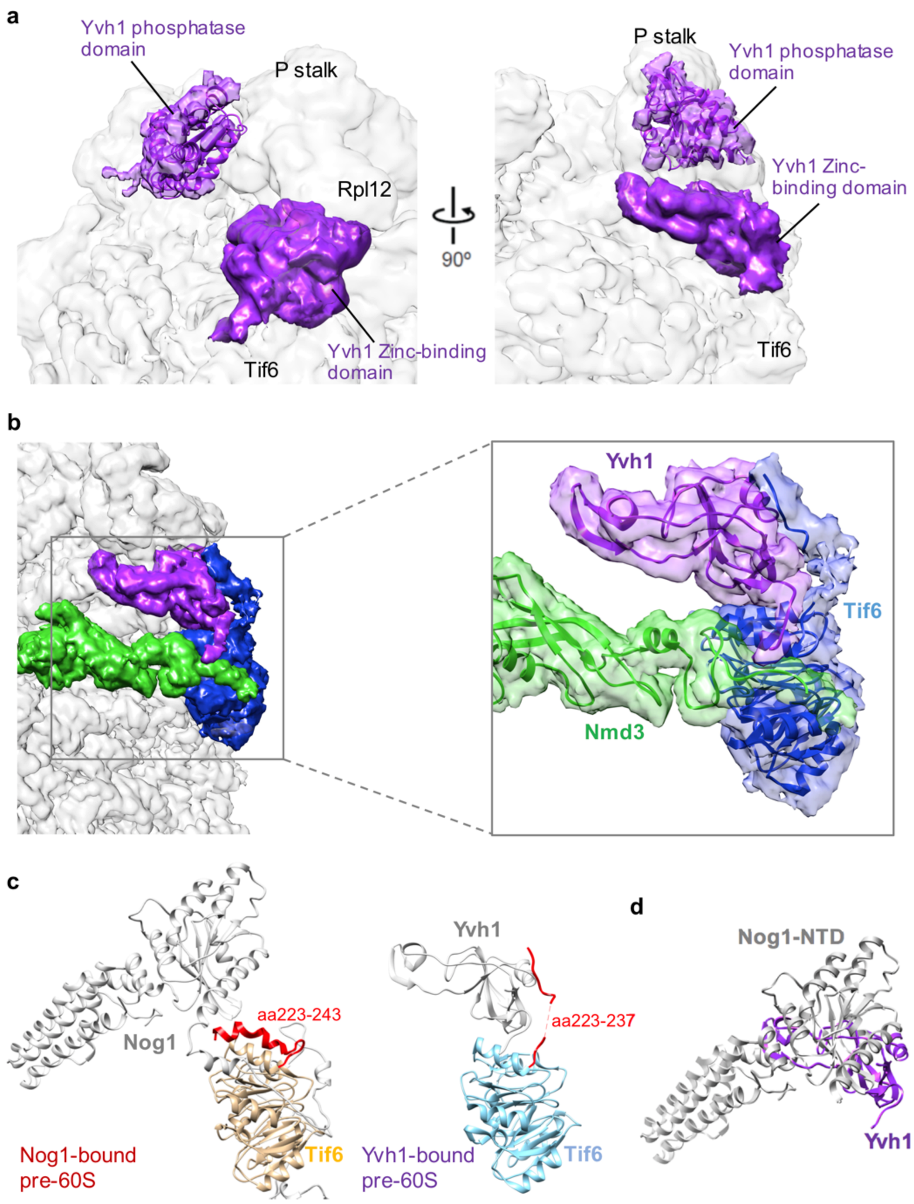

Supplementary Fig. 7 | Interactions among Tif6, Nog1, Nmd3 and Yvh1. a, The structure of the Yvh1 phosphatase domain from *Chaetomium thermophilum* (PDB: 5M43) was docked into a density observed at low threshold on the face of the P stalk facing the central protuberance. This roughly corresponds to the previously reported position of the phosphatase domain of Yvh1 [2]. **b,** Segmented densities for Yvh1, Nmd3 and Tif6 (left panel) with atomic structures modeled into the densities (right panel). At low threshold, the C-terminus of Tif6 can be traced as a continuous density extending to the surface of Yvh1, although it is hard to model the middle of Tif6 C-terminus. The extreme N-terminus of Nmd3 extends over Tif6 and is crossed by the C-terminus of Yvh1. **c,** Comparison of the position of the C-terminal tail of Tif6 (aa223-246) in the presence of Nog1 versus Yvh1. The tail folds back over the globular domain of Tif6 in the presence of Nog1 but repositions by rotating ~60° to extend to the surface of Yvh1 in the Yvh1-containing particles. **d,** Structural conflict of Nog1 and Yvh1 on pre-60S particles. Nog1 from the ECI particle and Yvh1 from the PL particle are overlaid.

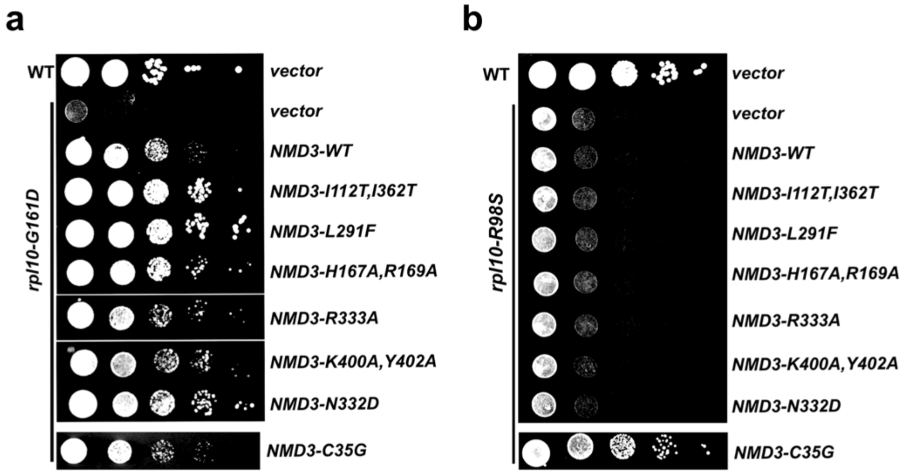

Supplementary Fig. 8 | Allele-specific suppression of *rpl10* mutants. a, Wild-type or *rpl10-G161D* mutant cells were transformed with empty vector or vectors expressing WT or mutant Nmd3, as indicated. Ten-fold serial dilutions of cultures were plated onto selective plates and incubated for 2 days at 30°C, a semi-permissive temperature *for rpl10-G161D*. Upper panel is reproduced from Fig. 4C for comparison. **b,** Wild-type cells were transformed with empty vector and P_{GAL1}-*RPL10* mutant cells were co-transformed with an *rpl10-R98S* expressing vector and vectors expressing WT or mutant Nmd3, as indicated. Ten-fold serial dilutions of cultures were plated onto selective plates and incubated for 2 days at 30°C, a semi-permissive temperature for *rpl10-G161D*.

Supplementary Table 1 | Statistics of cryo-EM structure determination and model refinement

**Data collection and processing**						
Sample	**Rlp24ΔC-TAP pre-60S**			**Nmd3-TAP pre-60S from diazaborine-treated cells**		
Microscope	FEI Titan Krios			FEI Titan Krios		
Direct electron detector	Gatan K2 Summit			Gatan K2 Summit		
Voltage (keV)	300			300		
Pixel size (Å)	1.1			1.1		
Total dose (e^-/ $Å^2$)	40			40		
Defocus range (µm)	1.0 - 2.5			1.0 - 2.5		
Total micrographs	7,403			6,179		
Used micrographs	6,860			5,839		
Total particles	326,527			485,448		
Particles for initial 3D refinement	178,310			340,712		
**Model building and refinement**						
Model name	**LN**	**ECI**	**ECL**	**PL**	**LE**	**RI**
	EMD-0369 PDB-6N8J	EMD-0370 PDB-6N8K	EMD-0371 PDB-6N8L	EMD-0372 PDB-6N8M	EMD-0373 PDB-6N8N	EMD-0374 PDB-6N8O
Map resolution at FSC 0.143 (Å)	3.5	3.6	3.6	3.5	3.8	3.5
Initial model used (PDB ID)	3JCT	3JCT	3JCT	5T62	5T62	5T62
Model composition						
Peptide chains	46	45	45	44	45	46
Residues	7,767	7,441	7,094	7,010	7,387	7,594
RNA chains	3	3	3	3	3	3
RNA bases	3,326	3,479	3,479	3,482	3,482	3,480
R.M.S. deviation						
Bond length (Å)	0.007	0.006	0.007	0.006	0.006	0.007
Bond angle (°)	0.880	0.866	0.879	0.814	0.862	0.848
Ramachandran plot						
Outliers (%)	0.33	0.42	0.46	0.38	0.50	0.40
Allowed (%)	7.30	6.58	7.32	6.54	6.78	7.28
Favored (%)	92.37	93.00	92.22	93.08	92.72	92.32
Validation (protein and RNA)						
Clashscore, all atoms	4.00	4.32	4.17	3.41	3.92	3.28
Molprobity score	1.66	1.66	1.68	1.58	1.64	1.59
Rotamers outlier (%)	0.51	0.51	0.64	0.43	0.48	0.56
Correct sugar puckers (%)	98.61	98.82	98.82	99.05	98.88	99.02
Good backbone conformations (%)	77.42	76.97	77.98	79.00	78.40	80.26

Supplementary Table 2 | Strains used in this study

Strain	Genotype	Source
BY4741 (WT)	*MATa his3Δ1 leu2Δ0 met15Δ0 ura3Δ0 met15Δ0*	[Open Biosystems TAP collection]
AJY1874	*MATa NMD3-TAP:: HIS3MX his3Δ1 leu2Δ0 met15Δ0 ura3Δ0*	[Open Biosystems TAP collection]
AJY1134	*MATα pep4-3 prb1-1122 ura3-52 leu2-3, 112 reg1-501 gal1*	[3]
AJY1657	*MATa ura3 leu2 rpl10-G161D*	[4]
AJY3370	*MATa* Nat^R ::P_{GAL1}-*RPL10 his3Δ1 leu2Δ0 ura3Δ0*	This Study

Supplementary Table 3 **| Plasmids used in this study**

Plasmid	Description	Source
pAJ123	*NMD3 LEU2 CEN*	[5]
pAJ415	*NMD3-L291F LEU2 CEN*	[4]
pAJ1334	*NMD3-I112T, I362T LEU2 CEN*	[4]
pAJ2726	*rpl10-R98S LEU2 CEN*	[6]
pAJ3020	*rpl10-R98S URA3 CEN*	[6]
pAJ3609	*NMD3-C35G URA3 CEN*	[6]
pAJ4305	*NMD3-H167A, R169A LEU2 CEN*	This Study
pAJ4307	*NMD3-R333A LEU2 CEN*	This Study
pAJ4308	*NMD3-K400A, Y402A LEU2 CEN*	This Study
pAJ4309	*NMD3-N332D LEU2 CEN*	This Study
pAJ3965	*GAL10::RPLP24-ΔC-TAP URA3 CEN*	This Study
pRS415	*LEU2 CEN*	[7]
pRS416	*URA3 CEN*	[7]

Supplementary References

1 Ben-Shem, A. *et al.* The structure of the eukaryotic ribosome at 3.0 A resolution. *Science* **334**, 1524-1529, doi:10.1126/science.1212642 (2011).

2 Sarkar, A., Pech, M., Thoms, M., Beckmann, R. & Hurt, E. Ribosome-stalk biogenesis is coupled with recruitment of nuclear-export factor to the nascent 60S subunit. *Nat Struct Mol Biol* **23**, 1074-1082, doi:10.1038/nsmb.3312 (2016).

3 Hovland, P., Flick, J., Johnston, M. & Sclafani, R. A. Galactose as a gratuitous inducer of GAL gene expression in yeasts growing on glucose. *Gene* **83**, 57-64 (1989).

4 Hedges, J., West, M. & Johnson, A. W. Release of the export adapter, Nmd3p, from the 60S ribosomal subunit requires Rpl10p and the cytoplasmic GTPase Lsg1p. *Embo J* **24**, 567-579 (2005).

5 Ho, J. H. & Johnson, A. W. NMD3 encodes an essential cytoplasmic protein required for stable 60S ribosomal subunits in Saccharomyces cerevisiae. *Mol Cell Biol* **19**, 2389-2399 (1999).

6 Patchett, S., Musalgaonkar, S., Malyutin, A. G. & Johnson, A. W. The T-cell leukemia related rpl10-R98S mutant traps the 60S export adapter Nmd3 in the ribosomal P site in yeast. *PLoS Genet* **13**, e1006894, doi:10.1371/journal.pgen.1006894 (2017).

7 Sikorski, R. S. & Hieter, P. A system of shuttle vectors and yeast host strains designed for efficient manipulation of DNA in Saccharomyces cerevisiae. *Genetics* **122**, 19-27 (1989).
