# Supplementary material for: Tightly-orchestrated rearrangements govern catalytic center assembly of the ribosome
Source: Nat Commun. 2019 Feb 27;10:958. doi: 10.1038/s41467-019-08880-0 (PMC6393466; doi:10.1038/s41467-019-08880-0)
Supplement: Supplementary file 3 — Description of Additional Supplementary Information [file 41467_2019_8880_MOESM3_ESM.pdf]

Description of Additional Supplementary Files

File Name: Supplementary Movie 1
Description: Architecture of 60S precursors. This movie shows each of the six atomic models displayed in Fig.1 rocked by 30-degrees around the y-axis twice. The Rpl12 structure is docked into ECI and ECL models.

File Name: Supplementary Movie 2
Description: Rearrangements of ribosomal proteins and RNA during maturation of the 60S. This video is an animation morphing between the structures in Fig 1. The Rpl12 structure is docked into ECI and ECL models.

File Name: Supplementary Movie 3
Description: Large-scale rearrangement of H89. This movie shows the large-scale rearrangement of H89 to its nearly-mature position where it engages with the histidine thumb of Nmd3 upon the release of the N-terminus of Nog1 from the A site.

File Name: Supplementary Movie 4
Description: Rotation of the NTD of Nmd3. This movie shows the 60-degree rotation of the NTD of Nmd3 upon Lsg1 engagement by interpolation between Nmd3 structures from the PL particle and the LE particle. The eL22 and eIF5A domains of Nmd3 remain relatively rigid.

File Name: Supplementary Movie 5
Description: Retraction of H38 by Rpl10. This movie shows the retraction of H38 away from the eIF5A domain of Nmd3 to adopt its mature position upon Rpl10 insertion.

File Name: Supplementary Movie 6
Description: Retraction of H89 by Rpl10. This movie shows the subtle (~10 Å) yet important retraction of the middle portion of H89 towards Rpl10, which drives H89 into its mature position to stabilize Rpl10 in its binding cleft between H38 and H89.
